# Global Longitudinal Strain Is Associated with Mortality in Patients with Multiple Myeloma

**DOI:** 10.3390/jcm12072595

**Published:** 2023-03-30

**Authors:** Zhu Cui, Francesco Castagna, Waqas Hanif, Samuel J. Apple, Lili Zhang, James M. Tauras, Ira Braunschweig, Gurbakhash Kaur, Murali Janakiram, Yanhua Wang, Yanan Fang, Juan C. Diaz, Carolina Hoyos, Jorge Marin, Patricia A. Pellikka, Jorge E. Romero, Mario J. Garcia, Amit K. Verma, Nishi Shah, Leandro Slipczuk

**Affiliations:** 1 Department of Internal Medicine, Montefiore Medical Center, Bronx, NY 10467, USA; 2 Cardiology Division, Montefiore Medical Center, Albert Einstein College of Medicine, Bronx, NY 10461, USA; 3 Oncology Department, Montefiore Medical Center, Albert Einstein College of Medicine, Bronx, NY 10461, USA; 4 Hematology Oncology Division, UT Southwestern Medical Center, Dallas, TX 75390, USA; 5 Division of Hematology, Oncology and Transplantation, University of Minnesota, Minneapolis, MN 55812, USA; 6 Department of Pathology, Montefiore Medical Center, Albert Einstein College of Medicine, Bronx, NY 10461, USA; 7 Clínica Las Americas, Medellin 50025, Colombia; 8 Heart and Vascular Center, Brigham and Women’s Hospital, Harvard Medical School, Boston, MA 02115, USA; 9 Department of Cardiovascular Medicine, Mayo Clinic, Rochester, MN 55902, USA

**Keywords:** multiple myeloma, Echo, global longitudinal strain, survival, ECG

## Abstract

Patients with multiple myeloma (MM) are at a high risk for developing cardiovascular complications. Global longitudinal strain (GLS) can detect early functional impairment before structural abnormalities develop. It remains unknown if reduced GLS is associated with reduced survival in patients with MM. We conducted a retrospective cohort analysis of patients diagnosed with MM between 1 January 2000 and 31 December 2017 at our institution. Patients with a 2D transthoracic echocardiogram completed within 1 year of MM diagnosis, left ventricular ejection fraction (LVEF) greater than 40%, and no history of myocardial infarction prior to MM diagnosis were included. GLS was measured using an artificial-intelligence-powered software (EchoGo Core), with reduced GLS defined as an absolute value of <18%. The primary outcome of interest was overall survival since myeloma diagnosis. Our cohort included 242 patients with a median follow up of 4.28 years. Fifty-two (21.5%) patients had reduced average GLS. Patients with reduced GLS were more likely to have an IVSd ≥ 1.2cm, E/E’ > 9.6, LVEF/GLS > 4.1, higher LV mass index, and low-voltage ECG. A Total of 126 (52.1%) deaths occurred during follow-up. Overall survival was lower among patients with reduced GLS (adjusted HR: 1.81, CI: 1.07–3.05).

## 1. Introduction

Patients with multiple myeloma (MM) are at a high risk of cardiac complications due to epidemiological factors (e.g., advanced age at disease onset), intrinsic disease characteristics (e.g., higher rates of anemia, chronic kidney disease (CKD) and cardiac amyloidosis), and treatment-related toxicities [1]. Early recognition of cardiac dysfunction in patients with MM might affect treatment selection and reduce cardiac toxicity from inappropriate dosing [2,3].

While two-dimensional echocardiograms (Echo) are widely accessible, early changes may be below the threshold for detecting structural abnormalities, leading to delays in appropriate care. Left ventricular (LV) global longitudinal strain (GLS) by speckle-tracking analysis of Echo images characterizes the degree of shortening in the longitudinal axis during systole and is thought to be a more sensitive marker than left ventricular ejection fraction (LVEF) for systolic dysfunction [4,5]. GLS can predict survival among patients with chronic ischemic cardiomyopathy, heart failure with reduced or preserved ejection fraction (EF), and light chain (AL) cardiac amyloidosis [6,7,8,9,10,11,12]. However, GLS measurement can be time-consuming and requires additional sonographer and physicians training. Recently, new developments in artificial intelligence (AI) introduced promising results for improved efficiency and reproducibility in GLS measurement [13,14,15,16].

Using AI-enabled GLS quantification, our study aims to investigate the prevalence and prognostic value of reduced GLS in patients with MM.

## 2. Methods 

### 2.1. Patient Cohort

We conducted a retrospective cohort analysis of patients diagnosed with MM between 1 January 2000 and 31 December 2017 at Montefiore Healthcare System. Patients were identified in our Cancer Registry database through Clinical Looking Glass (CLG) software. Patients with an Echo within one year after MM diagnosis and LVEF greater than 40% were included. Patients with a history of myocardial infarction (MI) prior to MM diagnosis were excluded. Those with monoclonal gammopathy of unknown significance, solitary plasmacytomas, and smoldering MM were excluded. Baseline demographic, comorbidity, laboratory, MM staging and treatment information were extracted through a combination of chart reviews and CLG software. Race was categorized as Black, Hispanic, White and other/unknown. Myeloma staging followed the conventional International Staging System (ISS) [17]. Revised ISS (R-ISS) was constructed based on the ISS, cytogenetic and lactate dehydrogenase (LDH) information available according to the criteria established by the International Myeloma Working Group (IMWG) [18]. Triplet therapy was defined as having received protease inhibitors (PI), immunomodulatory drugs (IMiD), and corticosteroids as first-line therapy, whereas doublet therapy consisted of corticosteroids with PI or IMiD. Treatment response was based on documentation in the outpatient oncology notes, which followed standard IMWG response criteria for minimal response, partial response, very good partial response, and complete response [19,20].

### 2.2. D Echocardiogram (Echo) Analysis

Echo images were obtained using conventional parasternal, apical and subcostal windows according to the standardized American Society of Echocardiography protocol [21]. Echo images were de-identified and transferred via a web-based system for analysis using EchoGo Core (v2.0, Ultromics, Oxford, UK). As previously described [13], the software automatically segments and contours the LV endocardial border and analyzes echocardiographic images using AI algorithms created by machine learning. Each patient had one Echo study analyzed for our study. If multiple Echos were performed for a patient, the one closest to the date of MM diagnosis was used for analysis. Patients whose Echo images were not analyzable by the software due to poor quality were excluded from the study (N = 34). EchoGo generated measurements of LVEF, LV GLS, and regional strain. The average GLS obtained from apical 4-, 2- and 3-chamber views was used for data analysis (triplane). When triplane data were not available, measurements obtained from 4- and 2-chamber views were used for analysis (biplane).

We validated the GLS measurements by EchoGo against manual measurements conducted using TomTec (TomTec, Unterschleissheim, Germany) and QLAB (Philips, Andover, MA, USA) in a separate validation cohort that consisted of 52 randomly selected oncology patients from our institution cancer patient database. In this validation cohort, all study patients received anthracycline-based chemotherapy between 2016 and 2019. Bland–Altman analysis was conducted to examine the systemic variation in the average GLS measurements between different analysis methods (Supplementary Figure S1). Inter-observer variation was also studied for each method.

Based on the cut-offs used in the literature and the Youden index, we used an absolute value of less than 18% as the cut-off for reduced GLS in our study [5]. The EF-strain ratio was calculated as the ratio of LVEF to GLS, using >4.1 as the cut-off for abnormality based on data from the literature [22]. Relative apical sparing (RELAPS) was examined by calculating the ratio of apical longitudinal strain to the sum of base- and mid-segment longitudinal strains, using >0.87 as the cutoff for the presence of RELAPS, as established by a previous study [22].

Other Echo parameters and the indication for obtaining Echo were extracted from the original Echo reports, generated by board-certified cardiologists. These included the following: the left atrial (LA) dimension, LV internal diameter during diastole (LVIDd), LV internal diameter during systole (LVIDs), interventricular septum thickness during diastole (IVSd), LV posterior wall dimensions (LVPWd), left ventricular mass index, LA volume by Simpson’s biplane method, tissue Doppler E/e’ (derived from the lateral wall), and stroke volume (SV). Indications for obtaining Echo cardiograms were classified into the following four categories: (1) symptoms: cardiac symptoms, abnormal ECG, or suspected cardiac amyloidosis; (2) screening before initiating myeloma treatment; (3) during myeloma treatment, including concern for treatment-induced cardiac toxicity; (4) other or unknown. 

### 2.3. Electrocardiogram (ECG) Analysis

ECGs obtained within one year of MM diagnosis were reviewed by board-certified cardiologists with electrophysiology training (J.E.R., J.C.D., C.H., J.M.). If multiple ECGs were available, the ECG closest to the diagnosis date was used. ECG intervals (PR, QRS, QT, RR, P wave duration) and QRS amplitude in all 12 leads were measured manually with electronic calipers. Conduction system disease including left anterior fascicular block (LAFB), right bungle branch block (RBBB), left bundle branch block (LBBB) or intraventricular conduction delay (IVCD)) was assessed using established diagnostic criteria [23]. PR interval >200 ms was considered to be indicative of atrioventricular conduction delay. Prolonged QTc was defined as greater than 483 ms regardless of sex, given the prior data showing correlation of this cutoff with survival in patients with cardiac amyloidosis [24]. Low voltage was determined by a QRS amplitude of <5 mm in all limb leads or <10 mm in all precordial leads. Left ventricular hypertrophy (LVH) was determined by Siegel criteria [25].

### 2.4. Mortality Ascertainment

Overall survival (OS) was defined as the time from MM diagnosis to death. Data were administratively censored on 31 December 2021. Mortality data were extracted by chart review. Patients who were lost to follow-up (no clinical encounter ≥ 6 months before the end of the administrative censor date) were contacted by phone to determine their mortality status. Those unable to be reached by phone with no documented death date were reported as being alive on the date of the last clinical encounter.

### 2.5. Statistical Analysis

Statistical analysis was performed using Stata 12 software (StataCorp, College Station, TX, USA). Normality of the data was assessed with the Shapiro–Wilk W test. For univariable analysis, a two-sample *t* test and Kruskal–Wallis test were performed for normally and non-normally distributed continuous variables, respectively, and the χ^2^ test was performed for categorical variables. Association between baseline variables and OS were analyzed using univariable proportional hazard Cox regression models. Variables significantly associated with OS were entered into the multivariable Cox regression model. Continuous variables that were not normally distributed were log-transformed for the Cox regression model. Proportional hazard assumption was tested using Schoenfeld residuals for all multivariable Cox regression models. The cumulative incidence of all-cause mortality was analyzed using Kaplan–Meier survival curves with a log rank test. The threshold for statistical significance was set as *p* < 0.05. Sensitivity analysis was performed given the potential confounding of unequal distribution of patients with mildly reduced LVEF (40–50%). This study was approved by the Albert Einstein College of Medicine institutional review board. 

## 3. Results

### 3.1. Patient Characteristics

**Figure 1:**
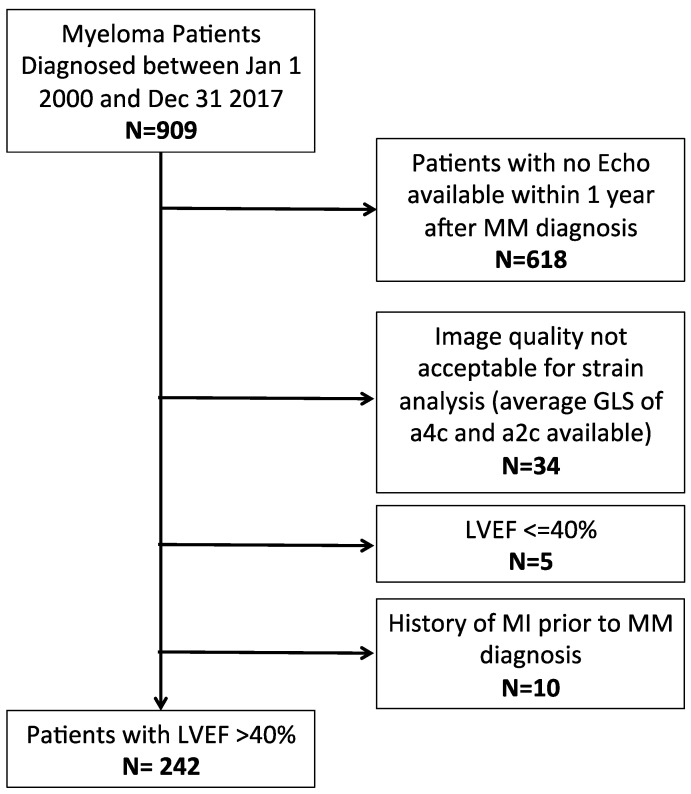
Patient selection flow diagram. GLS = global longitudinal strain; MM= multiple myeloma; LVEF = left ventricular ejection fraction.

Between 1 January 2000 and 31 December 2017, a total of 909 patients were diagnosed with MM at our institution (Figure 1). The final analysis cohort consisted of 242 patients. The median follow-up time was 4.28 years.

Fifty-two patients (21.49%) had an absolute GLS of less than 18%. As shown in Table 1, the group with reduced GLS included a lower percentage of women (23% vs. 52%, *p* < 0.001). There were no significant differences in the types of heavy and light chain involvement, R-ISS stages, ISS stages, or baseline lab values including median lactate dehydrogenase (LDH), beta 2 microglobulin, creatinine, and hemoglobin levels. The treatment types and treatment responses of patients with reduced versus preserved GLS are shown in Supplementary Table S1. We did not find any significant differences between the groups regarding the use of IMiDs and PIs as first-line treatment, use of induction chemotherapy, and exposure to medications with known cardiotoxicity such as doxorubicin and carfilzomib. Responses to first-line treatment and the percentage of patients who received autologous stem cell transplants were also similar between the two groups.

### 3.2. Echocardiographic and ECG Characteristics

The Echo and ECG parameters that compare patients with preserved and reduced GLS are shown in Table 2. A higher percentage of patients with reduced GLS underwent an Echo due to the presence of cardiac symptoms or abnormal ECG (63.46% vs. 40.53%). This included seven (13.46%) patients in the reduced GLS group who underwent an Echo due to clinical suspicion for cardiac amyloidosis, whereas only one patient (0.53%) in the preserved GLS group had such an indication. The average stroke volume and LVEF were lower among patients with reduced GLS (54.3 mL vs. 65.1 mL, *p* = 0.004; 53.97% vs. 64.21%, *p* < 0.001). The LV mass index was higher among patients with reduced GLS (*p* < 0.001). The prevalence of IVSd ≥ 1.2 cm and E/E’ > 9.6 was higher among patients with reduced GLS (*p* = 0.002 and 0.029). Nine patients (17.31%) in the reduced GLS group had an EF to strain ratio greater than 4.1, whereas no patient in the preserved GLS group had an increased EF to strain ratio (*p* < 0.001). No patient met criteria for the RELAPS in either group. While differences in the average diastolic LVID, systolic LVID, IVSd (as a continuous variable), and LVPWd also reached statistical significance, the absolute differences were small and unlikely to be considered as clinically significant. 

A total of 170 (70.3%) patients underwent an ECG within one year of MM diagnosis. The majority of patients (97.1%) were in the sinus rhythm. Conduction system disease was identified in 30 (17.6%) patients and 11 patients (6.5%) had a QTc > 483 ms. Low-voltage ECG was more prevalent among patients with reduced GLS (*p* = 0.049). LVH by Siegel criteria was present in 43 (23.9%) of patients. There was no significant difference in the prevalence of LVH on the ECG between the two groups (*p* = 0.80).

### 3.3. GLS and Overall Survival

**Figure 2:**
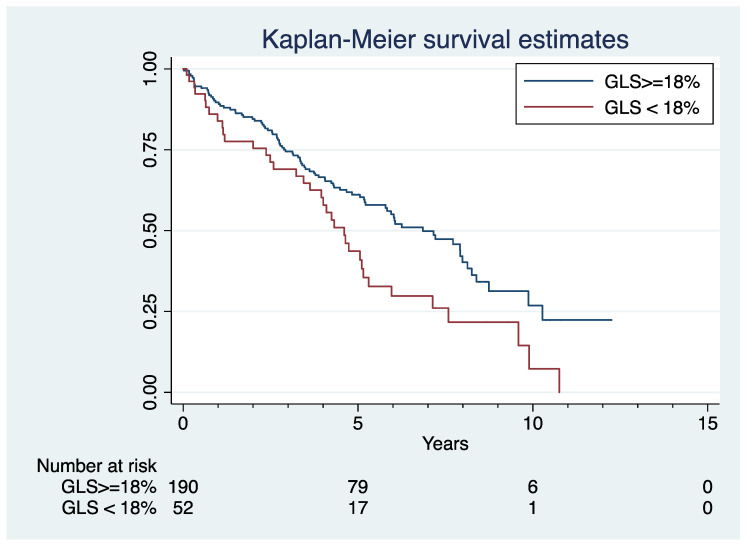
Global longitudinal strain and overall survival. Kaplan–Meier curves showing cumulative risk of all-cause mortality stratified by global longitudinal strain (GLS). Log-rank: *p* < 0.001.

There were a total of 126 (52.1%) deaths over a median follow-up time of 4.28 years. Median OS time was 3.98 years among those with reduced GLS and 4.31 years in those with preserved GLS (Figure 2; *p* = 0.010). GLS of <18% correlated with higher mortality hazard (HR: 1.65, CI: 1.12–2.43) in the univariable analysis. Hazard ratios of baseline demographic, MM, serum biomarkers, and selected Echo characteristics are shown in Table 3. Among the variables tested, diabetes, CCI, ISS, hemoglobin, creatinine and indication for Echo and LVEF were significantly associated with OS, and were included in the final adjusted Cox regression model. Despite more missing values, R-ISS instead of ISS was entered into the final model since it has been reported to offer prognostic value in MM [18]. LDH and b2-microglobulin were not entered into the model, as they were accounted for in R-ISS. Troponin was not tested, given the significant amount (64.3%) of missing cases. After adjusting for diabetes, CCI, R-ISS, hemoglobin, creatinine, indications for Echo and LVEF, GLS remained significantly associated with mortality (HR: 1.81, CI: 1.07–3.05; *p* = 0.026; Table 4).

### 3.4. Cases of Amyloidosis

Four patients in our cohort had confirmed amyloidosis via a biopsy of the colon or kidney. Three of the confirmed cases underwent an Echo for suspected cardiac amyloidosis and were also found to have reduced GLS. The other confirmed case of amyloidosis underwent an Echo for cardiac symptoms but was not found to have reduced GLS. Five other patients underwent an Echo for suspected cardiac amyloidosis but did not have biopsy proven disease, among them four patients had reduced GLS. We performed a sub-analysis excluding these patients and found similar results as prior (see Supplementary Table S2).

The patients who had Echo conducted due to the clinical suspicion of cardiac amyloidosis (N = 8) were included in the group with symptoms for analysis due to their small number. This group overall had a higher risk of death in the univariable analysis (HR: 2.07, 95% CI: 1.34–3.21; Table 3). In the final Cox regression model, patients who had underwent an Echo as a result of their symptoms had a higher hazard ratio for mortality, but this did not reach statistical significance (HR = 1.59, 95% CI: 0.96–2.64; Table 4).

### 3.5. Assessment of Confounding by LVEF

To assess whether the observed association between GLS and survival was confounded by the unequal distribution of patients with preserved versus mildly reduced EF, we conducted a sensitivity analysis by excluding patients with an EF < 50% (N = 16). Among the patients with an LVEF ≥ 50%, reduced GLS remained to be associated with mortality (HR= 2.00, CI: 1.17–3.45) after adjusting for the same co-variables listed above.

### 3.6. Assessment of Confounding by Treatment

To further assess potential confounding by treatment, we conducted a sub-analysis among patients whose Echo images were obtained within 3 months of MM diagnosis (Supplementary Table S3). In this cohort, 22 patients (22.9%) had reduced GLS, similar to the original cohort (21.5%). Reduced GLS continued to be significantly associated with mortality (HR: 2.09, CI: 1.10–3.95) in this cohort after adjusting for the same co-variables as above (diabetes, CCI, R-ISS, hemoglobin, creatinine and LVEF < 50%).

### 3.7. Assessment of Confounding by Selection Bias

To further assess potential confounding by selection bias, we conducted an analysis comparing the included and excluded cohorts (Supplementary Table S4). Overall, the two cohorts were similar. The median Charlson comorbidity index and myeloma stage distribution were similar. Average age was younger by 2 years in the study cohort. The study cohort had a higher percentage of Hispanic and lower percentage of White patients. However, there was more missing information among the patients not included in the study cohort.

## 4. Discussion

To the best of our knowledge, this is the largest study to date to examine GLS in patients with multiple myeloma. We report that reduced GLS correlated with worse OS among patients with MM independent of LVEF in the adjusted Cox regression survival analysis. This observation remained true when we excluded patients who had mildly reduced LVEF (EF 40–50%). Only two other publications examined GLS among patients with MM. The first investigated the association between GLS and survival rates among 115 patients receiving autologous stem cell transplants with high-dose melphalan conditioning [26]. Reduced GLS, defined as <17%, was found in 76% of the study cohort and correlated with survival rates at 1-year post-transplant. A second publication studied GLS prevalence in 40 patients with MM compared to 32 controls [27], and reported lower GLS among those with wall thicknesses >10 mm. Patients were only followed for six months in this study.

Reduced GLS is thought to be an early marker of cardiac dysfunction in conditions such as cardiac amyloidosis or drug toxicity [9,22,28]. This may be because GLS most directly reflects the contractile function of the longitudinal fibers, which are predominantly located in the sub-endocardium and may be first affected based on histology and magnetic resonance studies [9,29]. The cause of reduced GLS in our study cohort is likely to be heterogeneous. Undiagnosed cardiac amyloidosis, treatment-related cardiac toxicity, and new-onset ischemic cardiomyopathy are all potential causes of reduced GLS, among others. We categorized patients by indication for Echo into the following four categories: (1) conducted for the presence of cardiac symptoms; (2) conducted during myeloma treatment, including due to concerns of treatment-related cardiac toxicity; (3) conducted as screening prior to myeloma treatment initiation; (4) conducted for other or unknown reasons. We added indication as a variable in our adjusted Cox regression model to examine the effect of reduced GLS for patients with an Echo performed for a similar indication. Patients with reduced GLS had worse survival outcomes in this adjusted model.

Clinical and subclinical cardiac amyloidosis may be an etiology for the reduced GLS in our cohort. We observed a higher prevalence of IVSd ≥ 1.2 cm, E/E’ > 9.6, and low-voltage ECG among patients with reduced GLS, which are also features observed in cardiac amyloidosis [22]. Most interestingly, the prevalence of an increased EF to GLS ratio, one of the best Echo parameters for discerning cardiac amyloidosis from other causes of myocardial thickening, was significantly higher among patients with reduced GLS [22]. Unfortunately, we did not have biopsy data to confirm whether cardiac amyloidosis was present or not in patients with these Echo features. We were also unable to confidently exclude other forms cardiomyopathy, such as hypertrophic cardiomyopathy. Cardiac MRI had been shown to be useful in discerning various forms of cardiomyopathy, but these data were also lacking in our retrospective study [30,31]. Even with MRI imaging, the diagnosis of cardiac amyloidosis can be difficult. A recent study reported that late gadolinium enhancement (LGE) was likely to be a feature of more advanced disease and was not observed in around 35% of patients with AL cardiac amyloidosis [32]. Therefore, reduced GLS may serve as a useful benchmark to trigger additional workup for amyloidosis in myeloma patients, even if no reduction in LVEF or LGE was observed.

Another feature of our study is the use of AI-powered software for automated GLS analysis. Automated GLS measurement has been used for GLS analysis in a large multicenter cohort study that investigated Echo characteristics that correlated with in-hospital mortality among patients with COVID-19 [13]. In the same study cohort (WASE-COVID), automated GLS measurement was validated against manual quantification, and also found to reduce inter-observer variability [16]. AI-powered software also allows for higher throughput with less manual labor, making routine GLS assessment of Echo images a possibility.

Our study is limited by its small sample size, retrospective nature and limited number of confirmed extracardiac amyloidosis cases. Because of these limitations, we were unable to examine specific causes of death. Given the retrospective nature of our study, the potential bias associated with death adjudication and the competing comorbidities, we believe all-cause mortality is a stronger outcome. Furthermore, our study was underpowered to examine the differences in cause-specific death. Larger prospective studies with standardized, pre-specified protocols for categorizing causes of death would be necessary to answer the questions regarding GLS reduction and cause-specific death; however, in this case, all-cause mortality may still be a stronger outcome. While some patients with reduced GLS had additional echo features that were suggestive of cardiac amyloidosis, we did not have endomyocardial biopsy data or cardiac MRI data to further investigate the etiology for reduced GLS in these patients. Even with its limitations, our study is the first to report the association between reduced GLS and overall survival among myeloma patients. We think this observation is important to report and believe it will inform the design of future prospective studies that further elucidate the underlying mechanism of GLS reduction in this population and the benefit of routine GLS measurements on echocardiograms of myeloma patients as a gate-keeper for further testing with cardiac MRI or endomyocardial biopsy.

## 5. Conclusions

Reduced GLS is independently associated with reduced survival in patients with multiple myeloma. Our results demonstrate that GLS is a powerful tool that can be utilized to evaluate patients with MM who need additional attention during follow-up. Further research is needed to determine the underlying detected pathology and the value of additional comprehensive strain analysis, such as circumferential, radial and atrial strain.

## Figures and Tables

**Table 1:** Baseline demographics, comorbidities, MM, and lab characteristics.

	N	GLS ≥ 18%	GLS < 18%	*p* Value
Total	242	190 (78.51)	52 (21.49)	
Age (SD, years)	242	63.48 (10.59)	61.19 (12.10)	0.18
Female (%)	242	98 (51.58)	12 (23.08)	<0.001
Race (%)	242			0.42
Black		81 (42.63)	22 (42.31)	
Hispanic		65 (34.21)	14 (26.92)	
White		13 (6.84)	7 (13.46)	
Other/UK		31 (16.32)	9 (17.31)	
Hypertension (%)	242	84 (44.21)	21 (40.38)	0.62
Diabetes (%)	242	20 (10.53)	5 (9.62)	0.85
Median CCI (IQR)	242	2.5 (2)	3 (2.5)	0.92
Myeloma Type	242			0.35
Conventional		154 (81.05)	44 (84.62)	
Light Chain		33 (17.37)	6 (11.54)	
Non-Secretory		2 (1.05)	2 (3.85)	
UK		1 (0.53)	0 (0.0)	
Heavy Chain Involved (%)	242			0.40
IgG		105 (55.26)	34 (65.38)	
IgA		47 (24.74)	9 (17.31)	
Other/No heavy chain involvement		38 (20.00)	9 (17.31)	
Light Chain Involved (%)	242			0.65
Kappa		127 (66.84)	36 (69.23)	
Lamda		56 (29.47)	13 (25.00)	
Other		7 (3.68)	3 (5.77)	
R-ISS Stage	242			0.69
Stage I		10 (5.26)	5 (9.62)	
Stage II		68 (35.79)	19 (36.54)	
Stage III		22 (11.58)	5 (9.62)	
Missing		90 (47.37)	23 (44.23)	
ISS Stage	242			0.84
Stage I		47 (24.74)	16 (30.77)	
Stage II		48 (25.26)	13 (25.00)	
Stage III		58 (30.53)	14 (26.92)	
Missing		37 (19.47)	9 (17.31)	
Beta 2 Microglobulin (IQR, mg/L)	144	3.2 (3.6)	3.4 (2.4)	0.78
LDH (IQR, U/L)	150	195 (95)	203 (98)	0.62
Albumin (SD, g/dL)	220	3.7 (0.8)	3.7 (0.8)	0.82
Hgb (SD, g/dL)	220	10.2 (2.3)	10.6 (2.5)	0.29
Cr (IQR, mg/dL)	218	1 (1)	1 (1.5)	0.042
Troponin (IQR, ng/mL)	82	0.01 (0)	0.01 (0.02)	0.013

Abbreviations: GLS = global longitudinal strain; SD = standard deviation; CCI = Charlson comorbidity index; IQR = interquartile range; R-ISS = Revised International Staging System; LDH = lactate dehydrogenase; Hgb = hemoglobin; Cr= serum creatinine.

**Table 2:** Echo and ECG characteristics.

	N	GLS ≥ 18%	GLS < 18%	*p* Value
Total	242	190 (78.51)	52 (21.49)	
Time of Echo from MM diagnosis (IQR, days)	242	156 (195)	123 (193)	0.77
Indication for Echo:				0.040
Symptoms	110	77 (40.53)	33 (63.46)	
Screen prior to MM treatment	25	21 (11.05)	4 (7.69)	
During MM Treatment	36	31 (16.32)	5 (9.62)	
Other	71	61 (32.11)	10 (19.23)	
LA Dimension (SD, cm)	216	3.65 (0.55)	3.74 (0.72)	0.34
Diastolic LVID (SD, cm)	234	4.67 (0.62)	4.96 (0.71)	0.005
Systolic LVID (SD, cm)	214	3.03 (0.61)	3.52 (0.70)	<0.001
IVSd (IQR, cm)	234	0.94 (0.21)	1 (0.33)	0.016
IVSd ≥ 1.2 cm (%)	234	21 (11.54)	15 (28.85)	0.002
LVPWd (IQR, cm)	234	0.90 (0.2)	0.96 (0.19)	0.006
LV Mass Index (IQR, g/m2)	178	80.60 (24.48)	96.17 (29.90)	<0.001
EFSR ≥ 4.1 (%)	242	0 (0)	9 (17.31)	<0.001
LA Volume Index (SD, mL/m^2^)	57	29.21 (9.11)	30.78 (10.59)	0.62
E/E’ (IQR)	192	8.2 (3.7)	9.8 (6.5)	0.064
E/E’ >9.6 (%)	192	48 (30.77)	18 (50.00)	0.029
Stroke Volume (IQR, mL)	171	65.1 (22.3)	54.3 (18.5)	0.004
LVEF by Echo Go (SD, %)	242	64.21 (6.55)	53.97 (5.83)	<0.001
LVEF by Simpson’s Method (SD, %)	242	63.81 (8.85)	57.39 (8.99)	<0.001
Conduction Disease on ECG (%)	170	21 (15.91)	9 (23.68)	0.27
QTc > 483 ms (%)	167	7 (5.34)	4 (11.11)	0.25
LVH by Siegel Criteria (%)	170	34 (25.76)	9 (23.68)	0.80
Low-Voltage ECG (%)	170	0 (0)	2 (5.26)	0.049

Abbreviations: ECG = electrocardiogram; LA = left atrial; LVIDd = left ventricular internal diameter during diastole; LVIDs = left ventricular internal diameter during systole; IVSd = interventricular septum thickness during diastole; LVPWd = left ventricular posterior wall dimensions during diastole; EFSR = ejection fraction strain ratio; LVEF = left ventricular ejection fraction; LVH = left ventricular hypertrophy.

**Table 3:** Univariable analysis of baseline variables and OS.

	Hazard Ratio	95% CI	*p* Value
Age	1.02	1.00–1.03	0.071
Female	0.70	0.49–1.01	0.055
Race (Ref = Black)			
Hispanic	0.98	0.65–1.47	0.92
White	0.80	0.38–1.67	0.55
Other/UK	093	0.56–1.55	0.78
HTN	1.10	0.77–1.56	0.61
Diabetes	2.41	1.48–3.94	<0.001
CCI *	2.09	1.53–2.85	<0.001
ISS (Ref = Stage I)			
Stage II	1.88	1.14–3.08	0.013
Stage III	1.94	1.20–3.15	0.007
UK	1.23	0.69–2.19	0.478
R-ISS (Ref = Stage I)			
Stage II	1.14	0.54–2.45	0.72
Stage III	2.30	1.00–5.28	0.050
UK	1.00	0.48–2.12	0.99
Albumin	0.85	0.69–1.06	0.15
Hemoglobin	0.89	0.83–0.97	0.005
Creatinine *	1.59	1.25–2.04	<0.001
Indication for Echo (Ref = other)			
Symptoms	2.07	1.34–3.21	0.001
Screen prior to MM treatment	1.30	0.68–2.51	0.43
During MM Treatment	1.25	0.67–2.35	0.48
E/E’	1.66	0.96–2.86	0.070
IVSd ≥ 1.2 cm	1.59	0.99–2.56	0.054
LVEF	0.98	0.95–1.00	0.049
Stroke Volume	1.02	0.47–2.21	0.97
GLS < 18%	1.65	1.12–2.43	0.011

* Not normally distributed, was Log-transformed for regression modeling. CCI = Charlson comorbidity index; IVSd = interventricular septum in diastole; LVEF = left ventricular ejection fraction; GLS = global longitunidal strain.

**Table 4:** Multivariable Cox regression model for OS.

Variables Included	Hazard Ratio	95% CI	*p* Value
GLS < 18%	1.81	1.07–3.05	0.026
LVEF	0.99	0.96–1.02	0.41
History of Diabetes	1.73	0.97–3.11	0.065
CCI *	1.49	1.00–2.22	0.049
R-ISS Stages (Ref = Stage I)			
Stage II	1.56	0.71–3.41	0.26
Stage III	2.28	0.91–5.67	0.077
Stage IV	1.44	0.66–3.15	0.36
Hgb at Diagnosis	0.90	0.83–0.98	0.017
Cr at Diagnosis *	1.15	0.83–1.58	0.40
Indication for Echo			
Symptoms	1.59	0.96–2.64	0.074
Screen prior to MM treatment	0.87	0.40–1.90	0.74
During MM Treatment	1.07	0.54–2.12	0.86

* Not normally distributed, was Log-transformed for regression modeling.

## Data Availability

The data presented in this study are available upon request from the corresponding author. The data are not publicly available due to the inclusion of protected health information.

## Supplementary Materials

The following supporting information can be downloaded at: https://www.mdpi.com/article/10.3390/jcm12072595/s1. Table S1. Treatment for MM; Table S2. Sub-analysis excluding the patients with a diagnosis of extracardiac amyloidosis; Table S3. Sub-analysis of patients with echocardiogram within 3 months of MM diagnosis; Table S4. Baseline characteristics comparison of patients included vs not included in the final analysis; Figure S1. Validation of automated global longitudinal strain.
