# Supplementary material for: Global Longitudinal Strain Is Associated with Mortality in Patients with Multiple Myeloma
Source: J Clin Med. 2023 Mar 30;12(7):2595. doi: 10.3390/jcm12072595 (PMC10095531; doi:10.3390/jcm12072595)
Supplement: Supplementary file 1 [file jcm-12-02595-s001.zip › jcm-2239566-supplementary.pdf]

Supplementary Table S1. Treatment for MM.

	Total (N = 252)	GLS ≥ 18% (N = 193)	GLS < 18% (N= 59)	*p* Value
**1st Line Therapy (%)**				0.87
Triplet*	146 (60.33)	113 (59.47)	33 (63.46)	
Doublet**	81 (33.47)	65 (34.21)	16 (30.77)	
Missing	15 (6.20)	12 (6.32)	3 (5.77)	
**First-Line Use of IMiD (%)**	76 (31.40)	57 (30.00)	19 (36.54)	0.22
**First-Line Use of PI (%)**	211 (87.19)	167 (87.89)	44 (84.62)	0.37
**Induction Chemotherapy (%)**				0.99
At diagnosis	12 (4.96)	9 (4.74)	3 (5.77)	
Later in disease course	37 (15.29)	29 (15.26)	8 (15.38)	
No Chemo	176 (72.73)	139 (73.16)	37 (71.15)	
**Medication For Induction Chemotherapy (%)**				
Doxorubicin	35 (14.46)	28 (14.74)	7 (13.46)	0.82
Cyclophosphamide	41 (16.94)	30 (15.79)	11 (21.15)	0.36
Etoposide	37 (15.29)	28 (14.74)	9 (17.31)	0.65
**Maintenance Therapy (%)**				
Lenalidomide	128 (52.89)	101 (53.16)	27 (51.92)	0.87
Pomalidomide	23 (9.50)	19 (10.00)	4 (7.69)	0.62
Bortezomib	69 (28.51)	54 (28.42)	15 (28.85)	0.95
Carfilzomib	35 (14.46)	29 (15.26)	6 (11.54)	0.50
Ixazomib	13 (5.37)	12 (6.32)	1 (1.92)	0.21
Daratumumab	40 (16.53)	31 (16.32)	9 (17.31)	0.87
Elotuzumab	2 (0.83)	2 (1.05)	0 (0)	0.46
Panibinostat	2 (0.83)	2 (1.05)	0 (0)	0.46
**Response to 1st Line Therapy (%)**				0.41
CR/VGPR	59 (24.38)	43 (22.63)	16 (30.77)	
PR	102 (42.15)	85 (44.74)	17 (32.69)	
NR/PD	39 (16.12)	29 (15.26)	10 (19.23)	
Missing	42 (17.36)	33 (17.37)	9 (17.31)	
**ASCT Performed (%)**	175 (72.31)	140 (73.68)	35 (67.31)	0.36

* Triplet therapy was defined as having received protease inhibitors (PI), immunomodulatory drugs (IMiD), and corticosteroids as first-line therapy. ** Doublet therapy consisted of corticosteroids with PI or IMiD. ASCT = autologous stem cell transplant; CR = complete remission; GLS = global longitudinal strain; IMiD = immunomodulatory drug; MR = minimal response; PD = progression of disease; PI = protease inhibitor; PR = partial remission; VGPR = very good partial response.

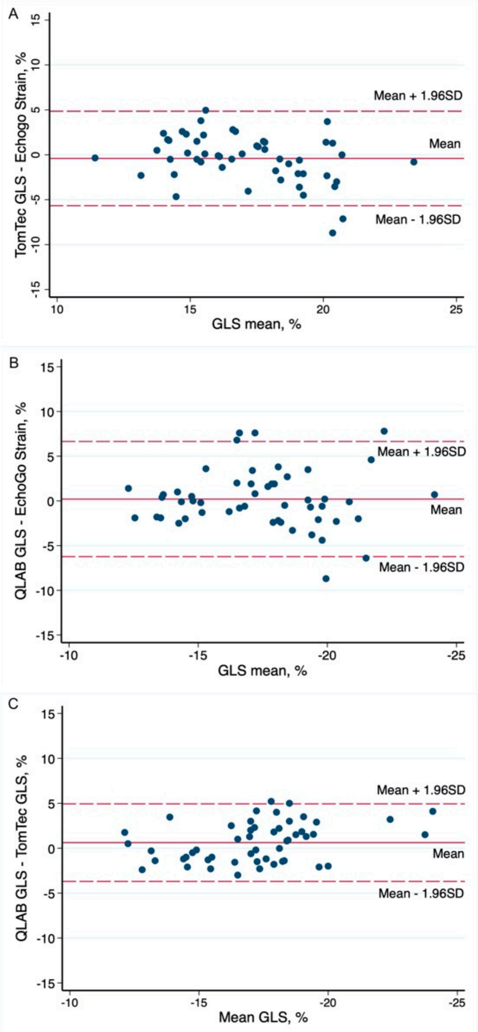

Supplementary Figure S1. Validation of automated global longitudinal strain. Bland–Altman analysis showed agreement between EchoGo longitudinal strain (GLS) and TomTec GLS (Figure S1A), between EchoGo GLS and QLAB GLS (Figure S1B) and between TomTec GLS and QLAB GLS (Figure S1C). Average absolute GLS was 17.3% (SD 3.3%), 16.9% (SD 2.4%), and 17.5 (SD 3.1%), respectively. Bland–Altman analysis showed bias of -0.4% ± 2.7% between EchoGo and TomTec measurements, and 0.2% ± 3.3% between EchoGo and QLAB measurements. The inter-reader correlation coefficient was 0.57 for TomTec and 0.71 for QLAB.

Supplementary Table S2. Sub-analysis excluding the patients with a diagnosis of extracardiac amyloidosis.

	Hazard Ratio	95% CI	P Value
**GLS <18%**	1.77	1.04–3.03	**0.037**
**LVEF**	0.99	0.96–1.02	0.41
**Diabetes**	1.80	0.97–3.34	0.062
**CCI***	1.46	0.98–2.18	0.062
**R-ISS (Ref = Stage I)**			
Stage II	1.59	0.72–3.49	0.25
Stage III	2.30	0.92–5.77	0.076
UK	1.48	0.67–3.25	0.97
**Hemoglobin**	0.90	0.83–0.98	**0.012**
**Creatinine***	1.13	0.82–1.57	0.45

Indication for Echo (Ref = other)			
Symptoms	1.64	0.98–2.74	0.059
Screen prior to MM treatment	0.89	0.41–1.94	0.77
During MM Treatment	1.08	0.54–2.15	0.83

* CCI and Cr are log transformed, given that they were not normally distributed.

Supplementary Table S3. Sub-analysis of patients with echocardiogram within 3 months of MM diagnosis.

**Univariable Analysis (N = 96, testing variables that were inputted into original model)**			
	**Hazard Ratio**	**95% CI**	**P Value**
**GLS <18%**	2.31	1.28–4.18	**0.005**
**Diabetes**	2.22	1.08–4.58	**0.031**
**CCI***	2.00	1.22–3.29	**0.006**
**R-ISS (Ref = Stage I)**			
Stage II	1.44	0.34–6.08	0.62
Stage III	2.03	0.45–9.15	0.36
UK	1.51	0.35–6.57	0.59
**Hemoglobin**	0.91	0.82–1.02	0.11
**Creatinine***	1.55	1.14–2.09	**0.004**
**LVEF**	0.97	0.94–1.01	0.15
**Indication for Echo (Ref = other)**			
Symptoms	1.81	0.87–3.74	0.11
Screen prior to MM treatment	0.82	0.27–2.48	0.73
During MM Treatment	0.82	0.10–6.48	0.85
**Multivariable Model (N = 96)**			
GLS <18%	2.13	1.16–3.88	**0.014**
Diabetes	1.67	0.77–3.65	0.19
CCI*	1.53	0.92–2.57	0.10
Cr*	1.43	1.04–1.98	**0.029**

* CCI and Cr are log transformed, given that they were non-normally distributed.

Supplementary Table S4. Baseline characteristics comparison of patients included vs not included in the final analysis.

	**Not Included in the Study Cohort (N = 667)**	**Included in Study Cohort (N = 242)**	**P Value**
**Age (SD, years)**	65.2 (12.1)	63.0 (10.9)	**0.014**
**Female (%)**	346 (51.9)	110 (45.5)	0.087
**Race (%)**			**0.048**
Black	285 (42.7)	103 (42.6)	
Hispanic	171 (25.6)	79 (32.6)	
White	93 (13.9)	20 (8.3)	
Other/UK	118 (17.7)	40 (16.5)	
**Median CCI (IQR)**	3 (2)	3 (2)	0.67
**Myeloma Type (%)**			0.92

Conventional	498 (81.0)	198 (82.2)	
Light chain	106 (17.2)	39 (16.2)	
Non-secretory	11 (1.8)	4 (1.7)	
**Myeloma Type Missing**	52 (7.8)	1 (0.4)	**<0.001**
**R-ISS Stage (%)**			0.77
Stage I	32 (11.5)	15 (11.6)	
Stage II	196 (70.5)	87 (67.4)	
Stage III	50 (18.0)	27 (20.9)	
**R-ISS Stage Missing (%)**	389 (58.3)	113 (46.7)	**0.002**
**ISS Stage (%)**			0.27
Stage I	147 (32.2)	63 (32.1)	
Stage II	168 (36.8)	61 (31.1)	
Stage III	142 (31.1)	72 (36.7)	
**ISS Stage Missing (%)**	210 (31.5)	46 (19.0)	**<0.001**
